# Impact of the COVID-19 pandemic on the epidemiology of severe burns: A single center study from a specialized burn center in Vienna

**DOI:** 10.1007/s00508-022-02149-1

**Published:** 2023-02-09

**Authors:** Alexandra Christ, Clement J. Staud, Matthias Wielscher, Annika Resch, Maryana Teufelsbauer, Christine Radtke

**Affiliations:** 1 Department of Plastic, Reconstructive and Aesthetic Surgery, Medical University of Vienna, Währinger Gürtel 18–20, 1090 Vienna, Austria; 2 Department of Dermatology, Medical University of Vienna, Vienna, Austria

**Keywords:** SARS‑CoV‑2, Burn trauma, Severe burns, Burn unit, Intensive care

## Abstract

**Introduction:**

Currently, very little detailed information on the epidemiological distribution and specificities of severely burned patients during the coronavirus disease 2019 (COVID-19) pandemic is available. This retrospective study aims to describe and compare this specific patient population based on 114 patients who were treated between March 2019 and March 2021 at the Center for Severe Burn Injuries at the Medical University of Vienna.

**Methods:**

To answer the research questions, a retrospective cohort study has been conducted over a period of 24 months, starting in March 2019 and ending in March 2021. To evaluate the epidemiological differences, the patients were divided into 2 observation periods of 12 months each.

**Results:**

In the period from 12 March 2020 to 11 March 2021, a total of 62 patients were admitted to the Center for Severe Burn Injuries. In comparison, only 52 patients were admitted in the same period of the previous year, which corresponds to an increase of 19.2%. In addition, it was noted that during the 2019–2020 observation period, 27% of patients were female and 73% male, whereas during the pandemic the gender distribution was 42% female and only 58% male. During the pre-pandemic observational period, 13 out of 52 patients admitted died (25%), whereas during the pandemic, 17 out of 62 patients succumbed to their injuries (27%).

**Conclusion:**

Although the severity of the COVID-19 pandemic seems to be decreasing, especially due to the increasing availability of vaccines, there is a need for more data on the impact of the crisis on severely burned patients. In contrast to the current literature, we have seen a greater number of inpatient admissions to the Center for Severe Burn Injuries, as well as significant differences in gender distribution. Our data also suggest that the circumstances of the pandemic have no influence on the likelihood of survival for patients with severe burns.

## Highlights

- The number of inpatient admissions increased by 19.2% during the pandemic.
- During the COVID-19 pandemic, there were major differences in gender distributions of patients.
- The pandemic does not seem to have an impact on mortality of severely burned patients.

## Introduction

The coronavirus disease 2019 (COVID-19) pandemic has challenged the healthcare system for almost 2 years now and affects the management and treatment of intensive care unit (ICU) patients worldwide. Specialized centers for severely burned patients are also confronted with various issues, both in terms of triage and treatment approaches. Recent literature prior to the outbreak of the COVID-19 pandemic described the epidemiological characteristics of severely burned patients, but very little detailed information on this special patient population has been published since the pandemic began. Two studies reported fewer inpatient admissions of critically burned patients due to the globally ordered lockdowns, but further data are limited at the present time. This retrospective study aims to address this limitation by recording epidemiological peculiarities in the context of the COVID-19 pandemic based on a relatively large patient population. Differences in burn severity, gender distribution, accident association and mortality are discussed, in order to clarify if the COVID-19 pandemic has had an impact on the overall number of inpatient admissions at the Center for Severe Burn Injuries. Furthermore, we want to investigate if the COVID-19 pandemic has had an impact on the epidemiological distribution of patient sex, burn severity and burn extent. The globally ordered lockdowns resulted in a drastic shift of everyday life to the domestic sphere, which is why this study also intends to highlight differences in the cause and context of the accident mechanisms of critically burned patients. Current data suggest that the risk of future pandemic diseases is constantly increasing due to ongoing globalization, livestock husbandry and growing populations [1, 2]. As the COVID-19 pandemic has demonstrated the drastic impact that a lack of preparedness can have on both the economy and global health, another aim of this study is to describe the relevance of adequately equipped specialized burn centers for potential future situations.

## Methods

A retrospective cohort study has been conducted over a period of 24 months, starting in March 2019 and ending in March 2021. To evaluate the epidemiological characteristics of severe burns during the pandemic, patients were divided into two observational periods. The first period covers all patients admitted to the Center for Severe Burn Injuries of the Medical University of Vienna between 12 March 2019 and 11 March 2020, representing the time before the outbreak of the COVID-19 pandemic. The second period includes all patients who were admitted between 12 March 2020 and 11 March 2021, representing patients admitted during the pandemic.

The data collection was carried out at the University Clinic for Plastic, Reconstructive and Aesthetic Surgery at Vienna General Hospital between May and June 2021. To ensure data protection, a password-protected, anonymized data table was created. Each patient was assigned a sequential number within this table.

The study includes all patients older than 18 years who were treated in the period from 12 March 2019 to 11 March 2021 in the context of an injury caused by flame, scalding, explosion, contact with solid matter, electric current, electric arc, acid and others in the Center for Severe Burn Injuries of the Medical University of Vienna. A standardized case report form based on WHO guidelines was used for data collection, including information on patient sociodemographics (age, gender), pre-existing health conditions, burn characteristics and etiology (total body surface area (TBSA), depth of the burn, affected body parts, cause of injury), admission and discharge date, length of stay at the Center for Severely Burned Patients, survival of the patients (yes/no), abbreviated burn severity index (ABSI) score of each patient and PCR test results for SARS-CoV‑2 virus. In cases of an incomplete data set, patients were not included in the study in order to ensure adequate results. Three patients who would initially have met the inclusion criteria were excluded due to this stipulation. A total of 114 patients were included in the study, of which only 1 patient had a positive PCR test result for the SARS-CoV‑2 virus at the time of admission.

The week of admission and the week of discharge from the ICU for each ICU patient before the COVID-19 pandemic (year 2019–2020) and during the COVID-19 pandemic (year 2020–2021) was recorded. For each week during the two intervals, we counted the number of patients in ICU care. From this, we calculated the number of patients per month and per year. These counts are plotted in Fig. 2 and 4. Next, we performed regression analysis using a generalized linear model, assuming a Poisson distribution of the ICU admissions per week data, to see whether or not the counts are dependent on the year of data recording. We performed a goodness of fit test, as implemented in the R‑package vcd [3] and calculated robust standard errors and *P*-values to control for mild violation of the distribution assumption. To evaluate the influence of sex on the ICU admissions we ran a generalized mixed model with and without sex as covariate. Subsequently, we performed a χ^2^-test to compare the two models.

Ethical approval was obtained from the Ethics Committee of the Medical University of Vienna in May 2021 prior to the data collection and statistical analysis. (#1422/2021).

## Results

**Fig. 1:**
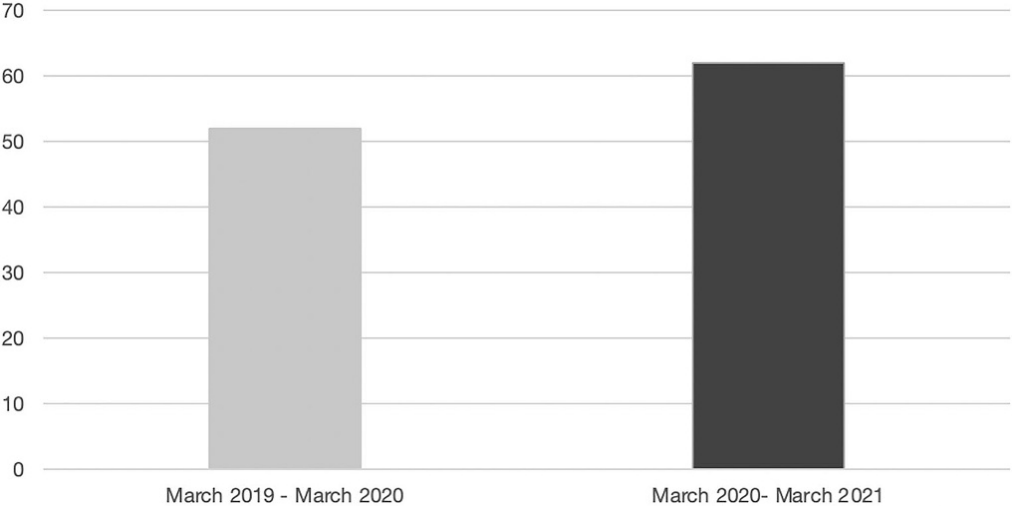
Overall number of inpatient admissions (*n* = 114); *x‑axis*: time period, *y‑axis*: total number of inpatient admissions

Figure 1 presents information on the number of inpatient admissions to the Center for Severe Burn Injuries at the Medical University of Vienna. In the first observation period, which represents the time before the outbreak of the COVID-19 pandemic, 52 patients were admitted, whereas in the second observation period starting with 12 March 2020 62 patients were treated as inpatients. This represents 19.23% more inpatient admissions than in the same period before the pandemic.

**Fig. 2:**
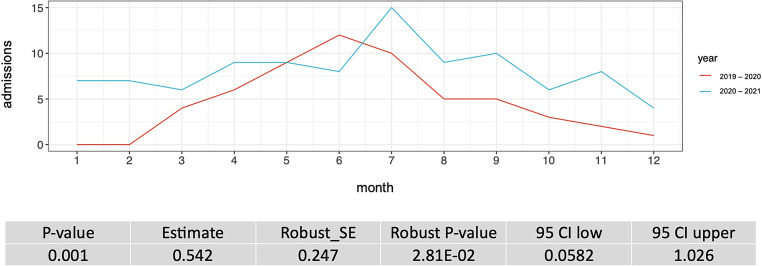
Intensive care unit (ICU) admissions over the course of the months; *x‑axis*: time in months (1 = January, 2 = February, 3 = ….), *y‑axis*: ICU admissions; *CI* confidence interval, *SE* standard error

Figure 2 demonstrates the absolute occupancy of the ICU over the individual months of the year and directly compares both observation periods. Based on this analysis, it can be concluded that the COVID-19 pandemic has a statistically significant impact on the overall number of inpatient admissions at the Center for Severe Burn Injuries.

**Fig. 3:**
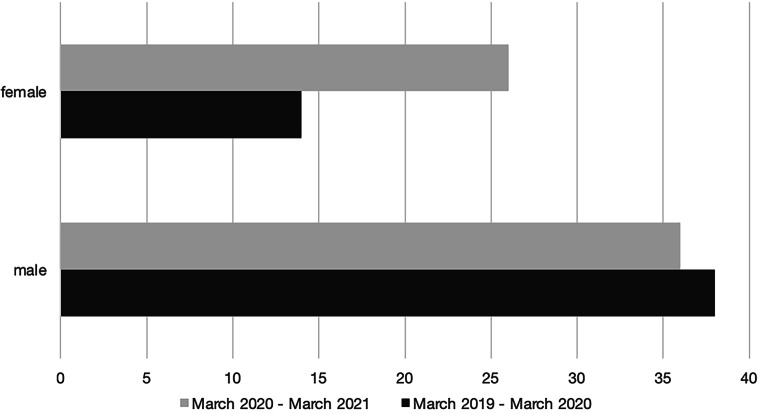
Sex distribution (observation period March 2019—March 2020: *n* = 52; male = 38, female = 14, observation period March 2020—March 2021: *n* = 62; male = 36, female = 26); *x‑axis*: total count of inpatient admissions, *y‑axis*: sex

**Fig. 4:**
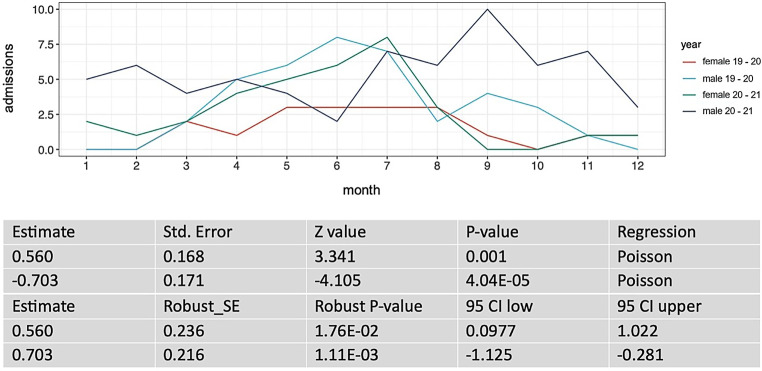
Sex distribution over the course of the months; *x‑axis*: months (1 = January, 2 = February, 3 = ….), *y‑axis*: occupancy of the ICU. *CI* confidence interval, *SE* standard error

Both observation periods were analyzed with respect to the sex distribution of the admitted patients at the Center for Severe Burn Injuries. Figures 3 and 4 represent those numbers. In the first period 27% (*n* = 14) of patients were female and 73% (*n* = 38) male, whereas during the pandemic the gender distribution was 42% (*n* = 26) female and only 58% (*n* = 36) male. Based on our findings, it can be concluded that the COVID-19 pandemic has a statistically significant impact on the epidemiological distribution of sex within our specific patient population. At this point in time, it is still unclear what the main contribution to this dynamic is.

**Fig. 5:**
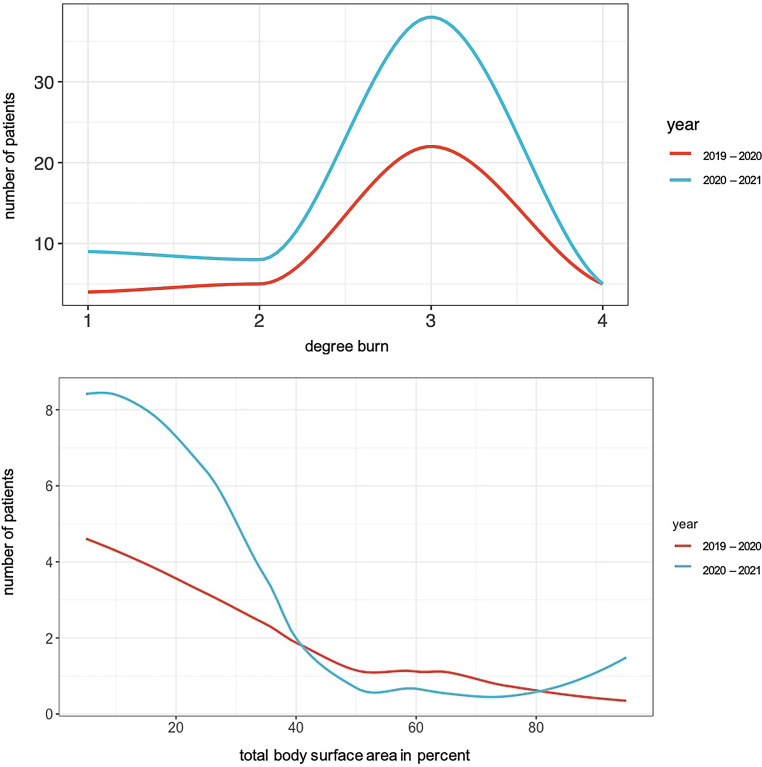
Comparison of burn severity (degree burn) and total body surface area (TBSA) in percent; *x‑axis*: degree burn/TBSA in percent, *y‑axis*: number of patients

It could be suspected that the patients were more seriously injured during the second observation period and therefore had to receive intensive medical care at the Center for Severe Burn Injuries for a longer period of time. To clarify this, both the burn severity (degree burn) and the burn extent (TBSA) of the patients during both periods were plotted and compared with each other (see Fig. 5). Apart from the fact that more patients were treated as inpatients between March 2020 and March 2021, it was found that the curves of both periods are almost identical. Based on this data, it can be concluded that neither burn severity nor extent changed as a result of the pandemic.

**Fig. 6:**
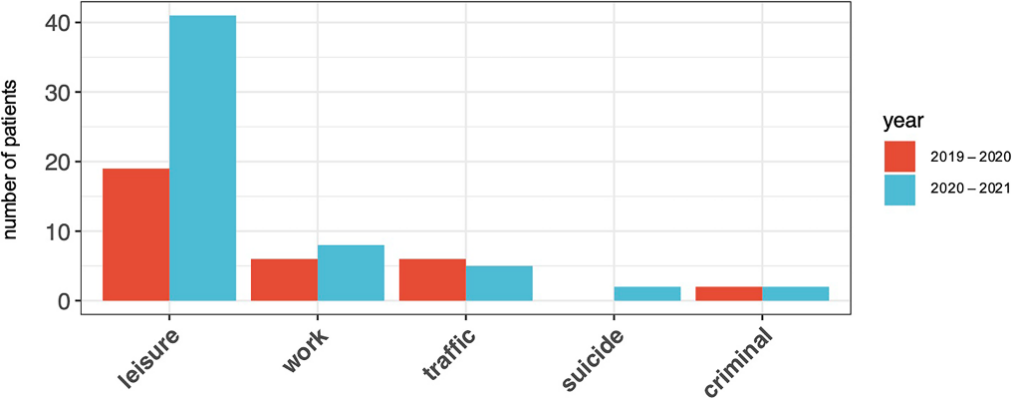
Context of accident in comparison; *x‑axis*: context of accident, *y‑axis*: number of patients

In order to clarify if the COVID-19 pandemic resulted in a change in the cause and context of the accident mechanisms of severely burned patients, a graphical representation of where the burn accident took place was created. Figure 6 represents our findings. Please note that almost all observed accidents within the category “leisure” occurred in domestic settings.

In the first observation period, 19 patients had an accident within their home environment, in comparison to 41 patients in the second observation period, which represents an increase of approximately 116%. A small increase in injuries in the work environment was also recorded but was not statistically significant in the context of the general increase in injury frequency and can therefore be neglected in relation to the research question. During the first observation period, there was no self-inflicted burn injury victim admitted to the Center for Severe Burn Injuries, whereas two suicidal burn accidents were recorded between March 2020 and March 2021.

## Discussion

Based on data from the relatively large patient cohort of 114 patients in total, it was determined that the number of inpatient admissions increased by 19.23% during the pandemic. This is in contrast to the results of other studies that addressed this topic, which noted a decrease in total admissions ranging from 7% to 49% during the pandemic [5–9]; however, a study conducted by Codner et al. reported relatively stable inpatient admissions over the course of the pandemic, as well as an increase in burns that required surgical treatment during shelter-in-place orders [4]. Two other studies showed that there was a significant increase in ethyl alcohol burns during the COVID-19 pandemic, due to increased use of disinfectants [20, 21].

In recent literature published prior to the pandemic, alcohol abuse has been linked to higher risk of severe burn injuries. One study even found that half of admitted burn patients were under the influence of alcohol at the time of the accident [16]. It is undisputed that the pandemic has had a serious impact on socioeconomic behavior patterns and the overall mental state of the population [22], and it has been reported that more than one in six adults increased their alcohol consumption during lockdown. This rise in alcohol consumption was associated with an increase in depressive symptoms as well as further decreased mental well-being [23]. Our findings highlight 2 cases of self-inflicted burns during the pandemic, in contrast to no recorded suicidal burns in the comparison group, which can potentially be explained by the overall decrease in mental well-being in society. The Austrian Health System Review from 2018 states that tobacco and alcohol represent the major health risk factors for adults living in Austria. Furthermore, the rate of alcohol consumption in Austria is among the highest in the EU [24]. As alcohol intake in those previously diagnosed with alcohol use disorders increased during the lockdowns, including in those who had been abstinent [25], it can be suggested that this partially explains the reported increase in total inpatient admissions at the Center for Severe Burn Injuries in Vienna, as compared to other international studies.

The Austrian Health System Review also states that average life expectancy in Austria is significantly above the EU average [24]. Multiple studies describe that people of advanced age are at higher risk for burn injuries, resulting in complications and mortality [26, 27]. Combining these factors, an increase in inpatient admissions in Vienna could therefore also be explained by admission of an above-average number of elderly people, who have been left without adequate care due to lockdown restrictions.

Furthermore, our study showed that the pandemic has had a direct influence on the number of female patients admitted. Current literature highlighting gender differences in severely burned patients during the COVID-19 pandemic addresses the possibility that there was an increase in injuries within the female population due to increased time spent in kitchen and dining areas during the globally ordered lockdowns [5]. The influence of gender on burns is much discussed in recent literature and offers significant room for discussion and further research. Some studies suggested gender-specific characteristics of burns in women, such as higher mortality after burn injuries, shorter hospital stays, and older age [10–12]. Other studies reported no differences in gender and burn severity and mortality [14, 15]. In the abbreviated burn severity index (ABSI) score, which is one of the most commonly used scores to assess burn injuries, one point is assigned for female gender [13]. A study conducted in South Africa in 2016 described that most severe burns are contracted by men at work, whereas women mostly suffered burns from accidents in the household [17]. Literature prior to the outbreak of the COVID-19 pandemic describes a fairly uniform distribution in terms of the gender of severely burned patients. On average, about 2/3 of severely burned patients are male and only 1/3 are female [10, 14, 15]; however, based on our results, these numbers do not reflect the gender distribution of admitted patients during the pandemic. As women usually sustain the severe burns in the home environment, it follows that more women are suffering from severe burn injuries as a result of lockdowns mandated around the world and the shift of daily life into the home.

While other studies report up to 4% of patients testing positive, we recorded only one COVID-19 positive patient [6]. Furthermore, no healthcare worker tested positive for the virus within the second observation period, which is noticeably fewer than what other recent literature reported [6, 18]. This is likely due the unique testing strategy in Austria, in which every patient must take a PCR test at the time of hospital admission, resulting in early isolation and interruption of the infection chain when the virus is detected. Although it currently seems as if the COVID-19 pandemic will change its dynamics due to the omicron variant, and that the disease will inevitably become endemic, we should learn from the available data how important the appropriate staffing and sufficient resourcing of specialized burn centers is [19].

## Conclusion

Although the severity of the COVID-19 pandemic seems to be decreasing, especially due to the increasing availability of vaccines and lower mortality rates of dominant mutant strains, there is a need for more data on the impact of the crisis on severely burned patients. In this single center study, we attempted to create an overview of the patient population of a specialized burn center during the ongoing COVID-19 pandemic. Therefore, we compared two equal observation periods (before the pandemic/during the pandemic) with each other. In contrast to the current literature, we have seen a greater number of inpatient admissions to the Center for Severe Burn Injuries, as well as significant differences in gender distribution. Our data also suggest that the circumstances of the pandemic have no influence on the likelihood of survival for patients with severe burns.
